# Abdominal pain with curious massive gastric ulcer: A case report of IgA vasculitis complicated with cytomegalovirus infection

**DOI:** 10.1097/MD.0000000000043050

**Published:** 2025-07-04

**Authors:** Yueyi Zhang, Aiming Yang, Qiang Wang

**Affiliations:** a Department of Gastroenterology, State Key Laboratory of Complex Severe and Rare Diseases, Peking Union Medical College Hospital, Chinese Academy of Medical Sciences and Peking Union Medical College, Beijing, China; b Department of Gastroenterology, The People’s Hospital of Xizang Autonomous Region, Lhasa, Xizang Autonomous Region, China.

**Keywords:** case report, cytomegalovirus infection, endoscopy, gastric ulcers, IgA vasculitis

## Abstract

**Rationale:**

Immunoglobulin A (IgA) vasculitis is a relatively rare autoimmune disorder in adults, typically affecting the skin, joints, kidneys, and gastrointestinal tract. It is often an overlooked cause of abdominal pain, and the diagnostic challenge is compounded when it coexists with infections. IgA vasculitis complicated by cytomegalovirus infection is a rare clinical scenario that presents significant challenges for diagnosis and treatment. This case is reported to raise awareness of the condition.

**Patient concerns:**

A 56-year-old female presented with abdominal pain, hematochezia, purpura, and reduced urine output. After corticosteroid treatment, her condition initially improved. However, 1 week later, the abdominal pain worsened, and gastroscopy revealed multiple large ulcers.

**Diagnoses:**

The diagnosis of IgA vasculitis with concomitant cytomegalovirus (CMV) infection was confirmed based on clinical and endoscopic findings, CMV-DNA titer changes, and the patient’s response to antiviral therapy.

**Interventions:**

Antiviral treatment for CMV was initiated, while corticosteroid therapy was gradually tapered.

**Outcomes:**

The patient’s abdominal pain significantly relieved, CMV-DNA levels decreased to normal, and follow-up gastroscopy showed a marked reduction in ulcer size.

**Lessons:**

During corticosteroid treatment for IgA vasculitis, if abdominal pain intensifies and multiple gastric ulcers are found on endoscopy, a high index of suspicion for an underlying infection, particularly CMV infection, is crucial. Raising awareness of this clinical scenario can improve diagnostic accuracy and enable prompt treatment adjustments.

## 1. Introduction

Immunoglobulin A (IgA) vasculitis is a multisystemic, immune-mediated disorder affecting the skin, joint, kidney, and digestive tract. In adult, its incidence is <15 per 1,00,000 individuals.^[1]^ The condition’s immune dysregulation mechanism and immunosuppressive therapy increase the risk of secondary infections, which can worsen the patient’s condition, complicate diagnosis, and impede treatment progress, presenting significant challenges.

Here, we report a rare case of IgA vasculitis complicated by cytomegalovirus (CMV) infection. We provide a detailed description of the diagnosis and treatment process, and hope to enhance awareness and improve the diagnosis and treatment of IgA vasculitis complicated with CMV infection.

## 2. Case presentation

A 56-year-old female presented with intermittent periumbilical colicky pain 1 month ago. Subsequently, she developed bloody stools accompanied by a decrease in urine output to <800 mL/d. No significant past medical history was reported. Physical examination revealed an anemic appearance, periumbilical tenderness, hypoactive bowel sounds and symmetrical lower limb edema; no ulcers in the mouth or genitals, and no rash of the limbs.

**Figure 1.**
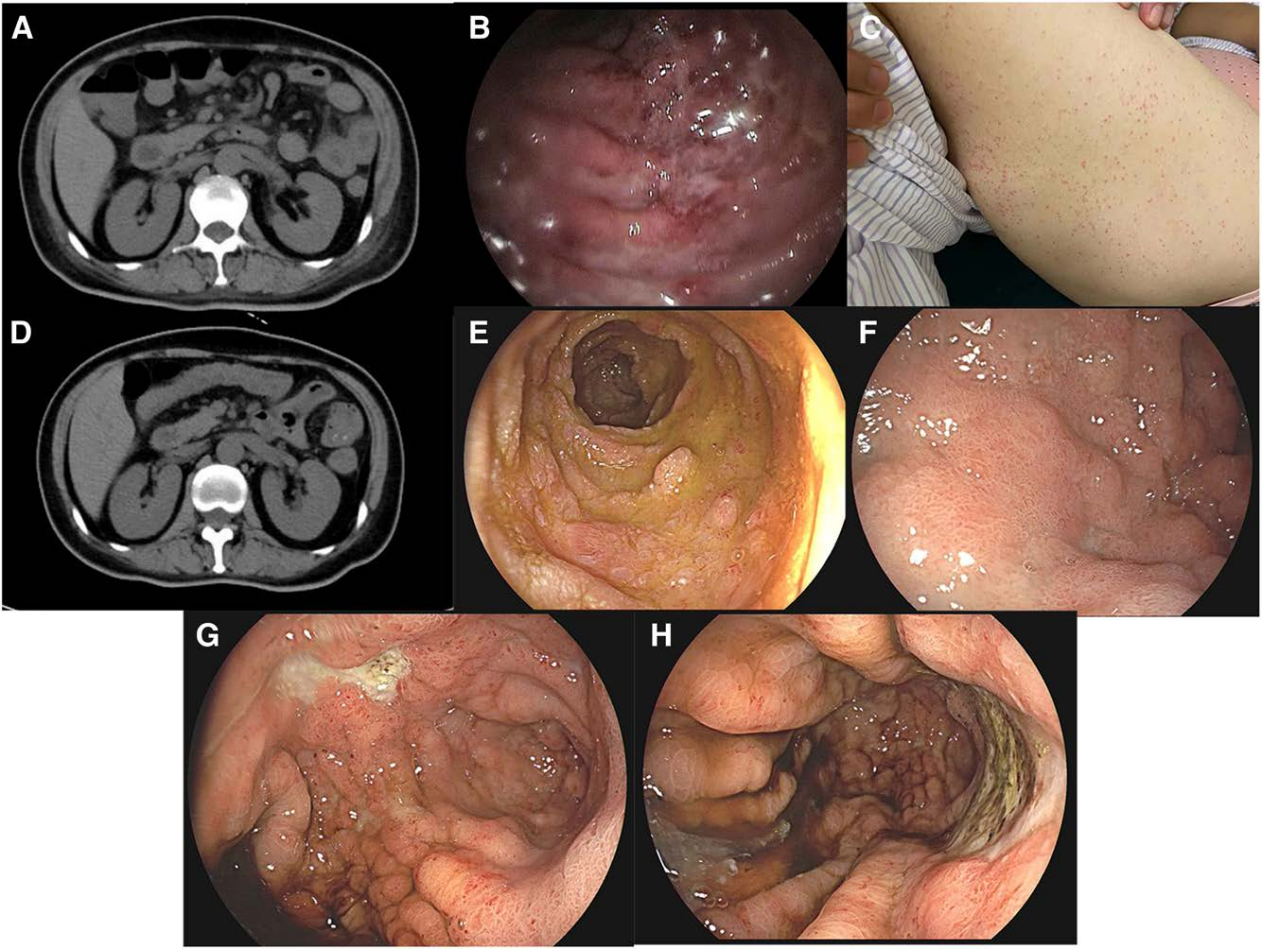
Radiologic and endoscopic imaging of the patient. (A) The initial abdominal contrast-enhanced CT revealed diffuse intestinal wall thickening; (B) the initial EGD revealed congestion, erosion, and bruising of the duodenal mucosa; (C) on the third day of hospitalization, the patient developed petechial rash on the front and inner thighs; (D) Following worsening abdominal pain, a subsequent CT scan demonstrated a notable improvement in intestinal wall edema; (E–H) subsequent EGD after exacerbation of abdominal pain showed reduced mucosal congestion but newly appeared multiple and extensive gastric ulcers. CT = computed tomography, EGD = esophagogastroduodenoscopy.

Laboratory examination showed elevated white blood cells (15.3 × 10^9^/L; reference 3.5–9.5 × 10^9^/L), severe anemia (hemoglobin 60g/L; reference 120–175 g/L), thrombocytosis (platelets 400 × 10^9^/L; reference 100–300 × 10^9^/L), and markedly elevated C-reactive protein (CRP) levels (140 mg/L; reference 0–6 mg/L). Biochemical tests indicated hypoalbuminemia (26 g/L; reference range: 30–45 g/L) with normal liver function. Renal function parameters including, serum creatine and estimated glomerular filtration rate, were within normal limits. Fecal test showed slightly elevated white blood cells (2/high-power field). Urinary analysis showed positive occult blood and 2 + urine protein, with a 24-hour total urine protein of 4.3 g (reference range: 0–0.2 g). Coagulation function, immunoglobulin and complement levels, gastrointestinal tumor markers and tuberculosis (T-SPOT) were within normal limits. Antinuclear antibody and anti-neutrophilic cytoplasmic antibody vasculitis auto-antibody tests were negative. Polymerase chain reaction assays for CMV-DNA and Epstein– Barr virus-DNA were negative, as were CMV antigen, anti-CMV immunoglobulin M (IgM), and immunoglobulin G in blood tests. Fecal tests for C. difficile toxin A/B were negative. Abdominal enhanced computed tomography (CT) showed diffuse intestinal wall thickening (Fig. 1A), and abdominal CT angiography was unremarkable. Esophagogastroduodenoscopy (EGD) revealed duodenal mucosal congestion, erosion, and bruising (Fig. 1B). Due to severe anemia and in line with the patient’s wishes, renal biopsy has been deferred. Despite nutritional support, antibiotics, and gastric mucosal protection treatments, the patient did not show improvement.

On the third day of hospitalization, the patient developed petechial rash on the front and inner thighs (Fig. 1C). Considering the comprehensive skin, kidney and digestive symptoms, we leaned towards diagnosing IgA vasculitis. Empirically, we initiated intravenous steroid therapy (methylprednisolone 80 mg/d), resulting in significant improvement in abdominal pain, decreased white blood cell count, and CRP levels. Renal function also improved, with the 24-hour urine protein decreasing to 0.96 g and urine output recovering to 1500 mL/d.

However, 1 week later, the patient’s abdominal pain worsened, along with decreased appetite, requiring parenteral nutrition support. Laboratory tests showed normal white blood cell count, with stable level of CRP and 24-hour urine protein. Follow-up abdominal CT and EGD showed improvement in intestinal wall thickening and mucosal congestion (Fig. 1D–F), but newly emerged multiple and massive gastric ulcers were detected during EGD (Fig. 1G, H). Suspecting a complicating disease, particularly opportunistic infections, we reevaluated infection indicators, including CMV-DNA, Epstein– Barr virus-DNA, stool culture, and C. difficile screening. Interestingly, CMV-DNA showed a significantly elevated titer of 5130 copies/mL (reference range: 0–400 copies/mL), confirming CMV infection. Biopsy of the ulcerative lesions revealed gastric mucosal inflammation, but viral inclusion bodies were absent, and both CMV immunohistochemistry and in situ hybridization were negative.

Ganciclovir was administered at 250 milligrams twice daily (5 mg/kg/d), alongside a reduction in methylprednisolone to 40 mg daily to alleviate immunosuppression. Subsequently, the patient experienced improved abdominal pain, diarrhea, and appetite. Two weeks after antiviral treatment, CMV-DNA became negative. Considering the patient’s initial presentation with both gastrointestinal and renal involvement, including extensive intestinal damage and a history of severe gastrointestinal bleeding, we added oral cyclophosphamide (50 mg/d) to enhance immunosuppressive therapy during steroid tapering, following a 3-week period of CMV-DNA negativity, to prevent relapse. The monitoring of 24-hour urine protein gradually decreased to 0.37 g, with no signs of CMV recurrence. Two months later, a follow-up EGD revealed a noticeable reduction in the size of gastric ulcers compared to earlier examinations.

## 3. Discussion

In this report, we describe a case of IgA vasculitis complicated by CMV infection, presenting with worsening abdominal pain during steroid therapy and the development of multiple massive stomach ulcers.

The diagnostic process was challenging. Initially, a delayed rash onset complicated the diagnosis of IgA vasculitis. Subsequent rash appearance, along with diffuse abdominal pain and renal signs, confirmed IgA vasculitis according to the 2010 EULAR criteria.^[2]^ Despite improvements in renal symptoms, the rapid deterioration of gastrointestinal symptoms and the emergence of new gastric ulcers during steroid therapy once again presented diagnostic challenges. Notably, a significant increase in CMV-DNA levels after steroid therapy, coupled with negative CMV-IgM, suggested an underlying CMV infection. Rapid improvement in gastrointestinal symptoms post anti-CMV therapy further supported IgA vasculitis complicated by CMV infection.

CMV is a ubiquitous virus belonging to the Herpesviridae family. The natural course can be categorized into 3 subtypes: primary infection, reactivation (following primary infection, immunity, and latency), and reinfection (superinfection with a new exogenous viral strain despite prior immunity). In immunocompetent individuals, CMV infection is often asymptomatic or presents as mononucleosis, though it may also cause end-organ diseases such as colitis and gastric ulcers.^[3]^ A retrospective study found that immunocompetent patients treated with glucocorticoid have a higher risk of developing CMV-related colitis.^[4]^ Additionally, in patients with intestinal diseases like ulcerative colitis, the risk of secondary CMV colitis is elevated, likely due to factors such as compromised intestinal mucosal barriers and impaired mucosal immunity.^[5]^ In this case, the patient was CMV-immunoglobulin G negative at admission but later tested positive for CMV-DNA, indicating a primary infection. The patient had no prior history of immunosuppressive diseases but developed CMV infection following IgA vasculitis and steroid treatment, presenting with CMV colitis and gastric ulcers. The mechanism likely involves significant gastrointestinal mucosal damage due to IgA vasculitis, which weakens local immune defenses, with the gastrointestinal tract being the primary target organ for CMV infection under steroid-induced immunosuppression.

Few cases of IgA vasculitis complicated by CMV colitis have been reported, and their coexistence poses a significant diagnostic challenge. The clinical presentations typically falling into 2 categories: 1 involves initial improvement after steroid therapy followed by later deterioration, as seen in this case^[6,7]^; the other category entails a poor response to steroids, marked by persistent abdominal pain and/or ongoing kidney function deterioration.^[8]^ Both scenarios highlight the need for further screening for potential infections. Endoscopically, there is a resolution of IgA vasculitis-related erosions and congestion, concurrent with the appearance of multiple ulcers in the stomach and duodenum.^[6,7]^ Diagnostic significance is ascribed to CMV-IgM titers, CMV antigen, CMV-DNA, and the identification of viral inclusions in histopathological examination. However, given the low detection rate of inclusions, the negative result does not definitively exclude the diagnosis of CMV infection. Treatment with ganciclovir is typically effective, leading to a notable improvement in abdominal symptoms after an appropriate course of antiviral therapy and gradual tapering of steroids.^[6–8]^ Notably, this case also emphasizes the importance of infection prevention in IgA vasculitis patients receiving steroid treatment. CMV has an incubation period of 1–7 days, and the patient developed primary CMV infection over 1 week after admission, indicating a hospital-acquired infection. This highlights the need for enhanced preventive measures, such as strict hand hygiene.

## 4. Conclusion

In conclusion, we presented a detailed case of IgA vasculitis complicated with CMV infection and summarized the features of similar cases. We hope the management details provided in our case contribute to a better understanding of such conditions.

## Author contributions

**Data curation:** Yueyi Zhang.

**Supervision:** Aiming Yang, Qiang Wang.

**Writing – original draft:** Yueyi Zhang.

**Writing – review & editing:** Aiming Yang, Qiang Wang.
